# Effects of Intraoperative Dexmedetomidine Infusion on Postoperative Pain after Craniotomy: A Narrative Review

**DOI:** 10.3390/brainsci11121636

**Published:** 2021-12-11

**Authors:** Nesjla Sofia Syrous, Terje Sundstrøm, Eirik Søfteland, Ib Jammer

**Affiliations:** 1 Department of Clinical Medicine, University of Bergen, 5021 Bergen, Norway; nesjla.syrous@gmail.com (N.S.S.); terje.sundstrom@helse-bergen.no (T.S.); eirik.softeland@helse-bergen.no (E.S.); 2 Department of Neurosurgery, Haukeland University Hospital, 5021 Bergen, Norway; 3 Department of Anaesthesia and Intensive Care, Haukeland University Hospital, 5021 Bergen, Norway

**Keywords:** dexmedetomidine, neurosurgery, pain, postoperative, acute pain, perioperative medicine

## Abstract

Craniotomy involves procedures with high incidences of postoperative pain. Dexmedetomidine, a highly selective a_2_-adrenoreceptor agonist, has been shown to be beneficial in neuroanaesthesia. The purpose of this narrative review was to assess the effect and safety of dexmedetomidine given intraoperatively during anaesthesia compared to placebo and demonstrate the effect on acute postoperative pain in adult patients undergoing craniotomy. Literature published from 1996 until 2021 were analysed through a search of PubMed, Medline and Embase. Randomised controlled trials investigating intraoperative administration of Dexmedetomidine with evaluation of postoperative pain were included. Medical Subject Headings terms and free-text words were used to identify articles related to the intraoperative use of Dexmedetomidine and postcraniotomy pain. Thirteen distinct randomized controlled trials with 882 recruited patients undergoing craniotomy were identified as eligible for final inclusion. Intraoperative administration of dexmedetomidine is associated with decreased postoperative pain and opioid consumption, and it assures haemodynamic stability. Dexmedetomidine is an efficacious adjunct in craniotomy in adults, showing benefits in reduction of postoperative pain and analgesic consumption. Dexmedetomidine also offers haemodynamic stability. However, widespread methodological heterogeneity of the papers prohibits a valid meta-analysis.

## 1. Introduction

Studies have reported that 40–84% of neurosurgical patients experience moderate to severe pain during the first postoperative days, despite liberal use of intraoperative opioids [1,2]. Pain is usually most pronounced during the first 48 h after surgery [3,4]. However, up to 50% may experience chronic headache for months [1,5,6,7]. Acute postcraniotomy pain is predominantly located to the area of incision and involves pericranial muscle and soft tissue. Inadequate analgesia and pain after neurosurgery may cause a series of adverse events, such as agitation, hypertension, increased intracranial pressure and postoperative intracerebral haemorrhage. This can prolong the hospital stay and, most importantly, increase patient morbidity and mortality [8,9]. Large amounts of intra- and postoperative opioids may provide effective analgesia and haemodynamic stability, yet may also cause delayed awakening, respiratory depression and postoperative nausea and vomiting (PONV) [10]. Measures to avoid or to reduce the use of opioid analgesia are therefore increasingly incorporated in the analgesic regimens. 

Dexmedetomidine (DEX), a highly selective a_2_-adrenoreceptor agonist, has unique characteristics in providing sedation and analgesia [11]. Due to its central sympatholytic action, DEX produces dose-dependent sedation, antinociception and anxiolysis, while decreasing intraoperative hypertensive and tachycardic episodes. Reportedly, DEX provides a cooperative form of sedation, in which patients easily wake up and comply with testing. This allows early neurological evaluation, which is highly desirable in the neurosurgical population of patients [12,13]. Moreover, DEX is a useful anaesthetic adjuvant as it decreases the demand for opioids, intraoperative anaesthetics and inhalation anaesthetics without suppressing the ventilation [14]. The analgesic effects of DEX are mainly mediated by its effects on a_2c_- and a_2a_-receptors located on neurons in the dorsal horn [15]. Activation of the a_2c_-receptor subtype in the spinal cord also seems to accentuate the analgesic actions of opioids by inhibiting the afferent transmission of nociceptive signals [12,15]. Taken together, DEX has a broad range of favourable characteristics and is a potentially attractive adjunct in neuroanaesthesia [12]. In this review, we examine the effect of intraoperative DEX on postoperative pain in patients undergoing craniotomy. 

## 2. Materials and Methods

### 2.1. Search Strategy and Inclusion Criteria

We conducted a literature search to identify relevant randomised controlled trials (RCTs) in PubMed, Medline and Embase between 1996 until October 2021. Medical Subject Headings (MeSH) terms and free-text words were used to identify articles related to the intraoperative use of DEX and postcraniotomy pain. The complete search string can be found in the Supplementary File. We also identified potential papers detected by reading through retrieved manuscripts.

To be eligible for this review, publications had to meet the following inclusion criteria:

1. Clinical RCT study design.
2. Patient groups >18 years of age.
3. Patients underwent elective craniotomy under general anaesthesia and were extubated immediately after surgery.
4. Intraoperative intravenous administration of DEX compared to any control group.
5. One or several of the following outcomes reported: postoperative pain intensity, opioid/analgesic consumption at the postoperative care unit, postoperative pain-scores and/or number of patients experiencing pain at the postoperative pain unit.
6. Descriptions of protocols for anaesthesia, analgesia and rescue analgesics.
7. Availability of full text in English.

There were no limitations as to the dosing or duration of the DEX regimens, whether administered as continuous infusion or as bolus pre- and/or intraoperatively. If a study used two different doses of DEX and no dose-response relationship was detected, we decided to combine both groups as a single treatment group. The final search was performed on 13th October 2021. References of related reviews and meta-analysis were screened for additional papers. The authors (N.S. and I.J.) searched and retrieved abstracts, screened them for relevance, retrieved full-text manuscript, and extracted data. Any controversy concerning study selection or data extraction was resolved by discussion and consensus with a third reviewer (E.S. or T.S.) if agreement was not achieved. 

### 2.2. Outcomes of Interest

The outcomes of interest were procedural success and postoperative pain intensity rated on a Verbal- or Numeric Rating Scale (VRS/NRS) or a Visual Analogue Scale (VAS) during postoperative care, including the number of patients with moderate or severe pain (VRS/NRS > 4, VAS > 5). If pain intensity in the post-anaesthesia care unit (PACU) and the post-surgical ward was measured at several points in time, the highest pain score within the defined time period was registered. Procedural success was defined as completion of the specific neurosurgical operation without serious intra- or postoperative complications that caused patients to withdraw or be excluded from the study. Other outcomes of interest involved cumulative morphine/analgesic consumption, patients with DEX-related side effects (e.g., intra- or postoperative bradycardia, hypotension) and procedure-related outcomes (e.g., hypertension, tachycardia). 

### 2.3. Data Extraction

Relevant data from the included trials were extracted, tabulated and entered on Excel sheets (Microsoft Excel 2016, Microsoft Corporation, Redmond, WA, USA). The following outcomes were collated for analysis: The American Society of Anaesthesiologists Physical Status score, physical status and age, exclusion criteria, administration of DEX before induction, interventions (loading dose/single bolus, continuous infusion rate of DEX and anaesthesia protocol), administration of DEX after induction and all outcomes of interest; pain intensity during the stay in PACU, frequency of patients experiencing pain and PACU analgesic consumption.

## 3. Results

### 3.1. Included Trials

**Figure 1:**
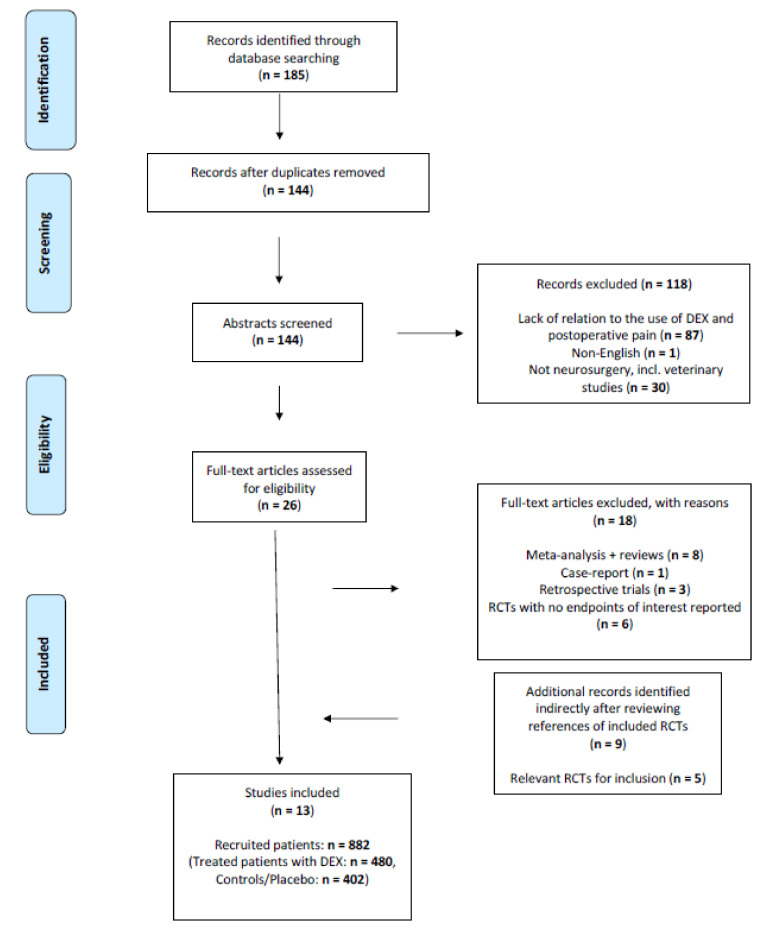
Flow diagram of study selection.

A total of 185 articles were identified from the literature search. After deduplication, title and abstract screening and identification of eligible trials for inclusion, a detailed full-text review was retrieved. Thirteen distinct RCTs with a total of 882 recruited patients undergoing craniotomy (480 treated patients with dexmedetomidine, 402 controls) were selected for final analysis. See Figure 1 for PRISMA flow chart of the included and excluded trials. Quality assessment of the included trials was performed after Grading of Recommendations Assessment, Development and Evaluation (GRADE) guidelines. The GRADE evidence profile of the included trials can be seen in Table S1 of the Supplemental Material.

Patients in the included studies were comparable as to age, sex, BMI, ASA-PS grade, comorbidity and planned neurosurgical procedure, further summarized in Table 1.

All study participants received anaesthesia intraoperatively according to a standard anaesthesia protocol, including infusion of the study drug or placebo. Some of the included trials had antiemetics, corticosteroids or analgesics as a part of the standard protocol as prophylaxis against PONV or pain, given before or at the time of endocranium closure. A majority of these trials had prophylactic measures for both PONV and postoperative pain. In three trials, ondansetron was given to all patients as PONV prophylaxis together with other substances as a part of a standard protocol; in one trial together with tramadol [18]; and in the two remaining trials ondansetron was given in combination with paracetamol against PONV and postoperative pain [2,26].

### 3.2. Pain Management

Two studies used VRS postoperatively for evaluating pain every 0, 2, 6, 12, 18, 24 and 48 h, or at 1, 5, 15 and 30 min in the PACU as soon as the patients were responsive to verbal stimuli. Postoperative analgesia was considered insufficient if the VRS score exceeded 8 or remained >4 for 15 min, and additional analgesic was administered as boluses [7,20]. Two trials used the NRS score to assess the pain intensity for 24 h postoperatively [2,18]. Six trials used VAS with pain scores above 4 or 5 points as thresholds to administer opioids. In four of these trials VAS was monitored every 5 min for the first 20 min then every 10 min for the rest of the time until the patients were discharged to the ward [17,21,22,24]. In the two remaining trials pain scores were assessed at 30, 60, 90 and 120 min, followed by 6, 12 and 24 h [25,26]. In one separate trial, the patient-controlled-analgesia was managed solely to provide opioids or other substances for patient analgesia and was discontinued when it was no longer needed by the patient [18].

### 3.3. Trials Reporting Opioid Consumption in the Post-Anaesthesia Care Unit

A majority of the included trials documented significantly less PACU opioid and analgesic consumption in the DEX groups as compared to the control groups [7,17,20]. Günes et al. reported eight additional patients in the control group with extra analgesic requirements [23], while Goettel et al. reported only three patients from the control group with the same requirements [21]. One trial also documented an increased time to first request of postoperative analgesia in the DEX group, in addition to the mean cumulative morphine consumption being significantly less in the same group [18]. Another trial reported that the control group required supplemental analgesia earlier than the DEX group (median time: 33 vs. 38 min) [19]. Tanskanen et al. compared the total amount of consumed oxycodone in the postoperative period with two different doses of DEX (D1 and D2) given as continuous infusions during surgery. As opposed to the majority of the trials, the results favoured the control group with the mean doses of oxycodone for postoperative analgesia being 5.6, 6.0 and 6.4 mg in the control-, D1- and D2-group, respectively [22]. Two trials reported no significant difference between the groups in the requirement of postoperative opioids and analgesics [16,24]. These studies were not powered to detect a difference in postoperative pain since this was a secondary outcome. Therefore, they may be unable to detect a significant difference in postoperative pain. Only one trial reported that no rescue drugs or postoperative opioids for pain management were needed in any of the groups [2].

### 3.4. Reported Pain Intensity in the Post-Anaesthesia Care Unit

All 13 included trials reported pain intensity in the PACU but in widely different manners. One trial reported PACU pain intensity as a number of patients experiencing significant pain. This specific trial used VRS to assess pain and concluded that 30 patients in the DEX group (total 88 patients) and six patients in the control group (total 45 patients) experienced no pain [20]. A total of three trials evaluated the PACU pain intensity as the amount of supplemental analgesia consumed, of which two trials found that patients assigned to DEX needed less opioids and analgesics, compared to the control groups [19,23], whereas one trial showed no difference in the postoperative opioids or antiemetics requirements [16]. The remaining nine trials reported PACU pain intensity by evaluating pain scores with VRS, NRS or VAS. In one trial pain intensity was reported by assessing VRS scores [7]. Two trials reported postoperative pain intensity by assessing NRS scores [2,18]. Furthermore, six trials reported PACU pain intensity by assessing VAS score for pain [17,21,22,24,25,26]. An overview of the reported effects of DEX on postoperative pain in the included trials can be seen in Table 2.

### 3.5. Safety and Side Effects

Side effects between the treatment and control groups were evaluated to estimate the safety of DEX administration during craniotomy. The consolidated results indicated that hypertension occurred more often in the control groups and that a lower incidence of cardiovascular variabilities was associated with the groups receiving DEX [20,21,24]. In most trials there were no significant differences in hypotension and bradycardia between the groups. However, three trials reported a decrease in systolic blood pressure and heart rate in the DEX group at different time points during surgery [17,21,23]. Intra- and postoperative tachycardia were generally more frequent in the control groups.

## 4. Discussion

In this review limited to and focused on craniotomies only, we found that the use of intraoperative DEX during craniotomy was associated with reduced postoperative pain and opioid consumption, constituting a safe alternative to opioid combinations. DEX-treated patients displayed fewer immediate haemodynamic responses that needed treatment. Liu et al. published a recent review on the effects of DEX on several neurosurgical procedures, including trans-sphenoidal procedures. They found a reduction in intra- and postoperative opioid consumption [27]. We have analysed postoperative opioid consumption together with different pain scores as surrogates for postoperative pain. Respectively, both studies show reductions in opioid consumption. We believe that our approach in outcome evaluation also reflects a broader and more pragmatic clinical routine.

### 4.1. Pain

Postoperative pain is one of the major causes of agitation and discomfort after neurosurgery [26]. Administering effective analgesia judiciously to prevent pain while avoiding opiate-related adverse events such as PONV and respiratory depression is one of the greatest challenges in the PACU [18]. Providing opioid-sparing and opioid-protective anaesthesia is therefore highly recommended [21]. Song et al. demonstrated reduced verbal NRS scores at 12 h postcraniotomy and prolonged time to first request for postoperative analgesia in addition to less opioids to control postoperative pain in the group receiving intraoperative DEX [18]. Similarly, Yun et al. reported that patients receiving infusions of DEX intraoperatively had significantly decreased NRS scores. In the same trial, the group with the highest bolus dose of DEX was associated with a lower incidence of moderate-to-severe postoperative pain as compared to the group receiving the lowest bolus dose of DEX and the control group [20]. Similar results were detected in a trial by Zheng et al. where the VAS scores for pain in both DEX groups were significantly reduced compared to the control group [25]. The improved analgesic and antinociceptive effects provided by DEX are described as resulting from inhibiting the up-regulation of pain signals by activating the posterior horn of the spinal cord and modulation of sympathetic response by releasing catecholamines [25].

DEX has also proven useful in other areas of surgery. A study by Arain et al. evaluating postoperative outcomes after major inpatient surgery showed a consistent reduction of opioid requirements by 30–50% in the group receiving DEX [28]. Xin et al. described that intraoperative infusion of DEX during laparoscopic cholecystectomy can alleviate postoperative delirium in elderly patients with mild cognitive impairment [29]. Furthermore, a systematic review by Halpin et al. revealed a decrease in postoperative delirium in patients undergoing cardiac surgery receiving postoperative DEX in comparison with commonly used sedatives and analgesics [30]. This emphasizes that DEX as an anaesthetic adjuvant is not only beneficial in reducing postoperative pain but also effective in preventing postoperative delirium when used as an adjuvant both intraoperatively and postoperatively [29,30,31].

However, in one study where DEX was compared to remifentanil as anaesthetic adjuvants, it was noted that DEX may not offer sufficiently desired effects during all stages of a craniotomy, and therefore it may not fully replace opioids. Administering a low-dose remifentanil infusion in addition to DEX may potentially achieve successful pain control [21]. This conclusion was not accomplished in the remaining included trials having compared DEX against intravenous remifentanil; in three of these trials, craniotomy patients receiving intraoperative DEX had significantly less pain and haemodynamic adverse events in the PACU, while the group receiving remifentanil required supplemental analgesia earlier than the group receiving DEX [19,21,23]. Differences in postoperative pain and management of analgesics are likely amplified due to the extreme short duration of action and half-life of remifentanil, provoking hyperalgesia [17,32]. DEX has an onset-of-action of approximately 15 min after intravenous administration. It has a distribution half-life of 6 min in adults and an elimination half-life of between 2.0 and 2.5 h [17]. In contrast, remifentanil has a half-life of about 3 min and an elimination half-life between 12 and 30 min [17]. Due to this, it is more likely that patients receiving remifentanil require analgesics immediately after surgery and an increased total amount of analgesics as compared to the group receiving DEX.

DEX has been shown to reduce opioid requirements [11,12,28]. It is believed that the primary mechanism of analgesic action of DEX results from inhibition of substance *p* release [26]. This is thoroughly explained by Bajwa et al. in which they report the analgesic effects of DEX being mainly mediated by a_2c_- and a_2a_-receptors located on neurons in the dorsal horn, by inhibiting the pro-nociceptive transmitters, primarily substance *p* and glutamate, and by hyperpolarization of spinal interneurons [15]. The most important of these sites may be the spinal cord, where the activation of a_2c_-receptor subtype seems to accentuate the analgesic actions of opioids in attenuating the transmission of nociceptive signals to brain centres [12,15]. However, the precise mechanisms and pathways by which it induces analgesia have not been fully discovered as there may be other mechanisms. This may explain the effect of DEX during craniotomy sincesome sensitive fibres spread from the trigeminal nuclei and not from dorsal horn neuron activity.

While remifentanil is rapidly metabolized and facilitates quick awakening, higher doses of fentanyl may cause delayed awakening and respiratory depression [33]. In one trial by Sriganesh et al., DEX was compared to intravenous fentanyl as a control. The authors found decreased NRS scores in the PACU in both the DEX and control group, with none of the groups requiring additional opioids [2]. Both fentanyl and DEX have short half-lives, though they are still longer than the half-life of remifentanil. This might explain why both fentanyl and DEX potentiate effective multimodal postoperative analgesia when used intraoperatively [2]. However, increased use of opioids could affect the recovery due to respiratory complications, delay the neurologic assessment, and lead to a higher incidence of PONV, whereas DEX as an adjuvant achieves a good analgesic profile without having any deleterious effects [26]. DEX and opioids have different mechanism of action in different areas of the brain. It may be of importance to combine both drugs for pain treatment to maximize analgesic effects while minimizing possible side effects.

### 4.2. Haemodynamic Quality

DEX has been associated with bradycardia and hypotension. This may predispose patients to cerebral ischemia [19,22]. DEX, being a central a_2_-agonist, causes a dose-dependent reduction in the sympathetic output from the brain, which may explain the haemodynamic findings in patients receiving this anaesthetic [34]. However, it was apparent from most trials that the cardiovascular stability was better maintained in DEX-treated patients [22]. In one trial where DEX was compared to saline, none of the patients in either group required treatment for bradycardia [7]. The same results were achieved by Song et al., reporting no significant difference in bradycardia and hypotension during the study period between the DEX group and the control group [18]. A possible explanation to these results is that the patients included in these trials received a relatively lower dose of continuous infusion of DEX without a bolus dose [7] or a relatively low initial loading dose followed by a continuous infusion [18]. This shows that bradycardia can be attenuated by administering a lower dose of DEX for continuous infusion rather than a larger dose or faster bolus(es).

### 4.3. Cost-Effectiveness

Evidence on the cost-effectiveness of intraoperative DEX on postoperative pain is lacking. A few studies have evaluated the cost-effectiveness of DEX on sedation in ICU patients finding benefits due to shorter ventilation days and shorter extubation times [35,36]. However, the focus in healthcare should be on the value of care for the patient [37]. In a neurostimulator implantation study by ter Bruggen et al. [38], the sedation costs of intraoperative use of DEX were more expensive when compared to Propofol. Nevertheless, the sedation costs represented less than 0.5% of the whole cost for the procedure, with the cost difference being significant but factually small. Use of DEX therefore added value for the patient (higher patient satisfaction, better awakening conditions) and was justified [39]. The cost-effectiveness of DEX in craniotomy patients should be a future health–economic research topic.

### 4.4. Limitations

The current review has a number of limitations. Trials did not include measuring a target plasma level concentration of DEX. Instead, a constant dosage regimen was selected which might have led to individual variations in patients due to inherent differences in drug metabolization. Furthermore, in several trials, patients received prophylactic drugs against postoperative pain and-/or PONV at the end of the surgical procedures. This might have affected levels of postoperative pain and analgesic consumption, making it difficult to assess the sole effects of DEX on postoperative pain per se. All studies had various intraoperative treatment protocols, including different administration methods and postoperative analgesia protocols. Variable times and methods of measuring pain among the trials, including different combinations of opioids with variable duration of actions, made the outcome measures of the included trials highly variable and challenging to compare. The main focus of this review was the effect of intraoperative DEX on postoperative pain. Relevant articles discussing pain only as a secondary outcome may therefore have been overlooked or not found by the search string.

There is no universal definition of the term “pain” and “experience of pain”. How to interpret pain is therefore a subjective matter, in addition to the subjectivity of pain assessment techniques. In this review pain was assessed by evaluating the postoperative pain scores, opioid/analgesic consumption and by looking at the number of patients experiencing pain between the study groups. However, due to heterogeneity of the included trials, especially in definitions and interpretations of pain, it was not possible to quantify the reviewed results from the different publications in a statistical manner [40]. One RCT was excluded for not being in English. It is unlikely that the results in this trial would change the overall outcomes presented in this review.

## 5. Conclusions

We found that intraoperative infusion of DEX during craniotomy is associated with decreased postoperative pain and analgesic consumption, with improved pain scores. In addition, DEX did not compromise hemodynamics among patients undergoing elective craniotomy. However, there were significant heterogeneities in treatment protocols. Future research and clinical implementation of DEX as an anaesthetic adjuvant is needed, emphasising better standardization of intra- and postoperative anaesthesia protocols.

## Figures and Tables

**Table 1:** Characteristics of patients receiving dexmedetomidine or placebo in the included trials. Values are numbers (proportion).

References	Country	ASA	Age (y)	Type of Surgery	DEX (n)	Control/ Placebo (n)	Bolus	Continuous Infusion (Per Hour)	Anaesthesia Protocol
Bekker, 2008 [16]	USA	I-II	18–65	Craniotomy (resection of brain tumour)	28	28	1. DEX: 1 μg.kg^−1^ 2. 0.9% Saline: 1 μg.kg^−1^	1. DEX: 0.5 μg.kg^−1^ 2. 0.9% Saline: 0.5 μg.kg^−1^	Propofol + Vecuronium + Fentanyl + Sevoflurane + Remifentanil
Peng, 2015 [7]	China	I-II	18–65	Supratentorial craniotomy	38	38	NO BOLUS	1. DEX: 0.5 μg.kg^−1^ 2. Saline: 0.5 μg.kg^−1^	Propofol + Fentanyl + Cisatracium + Sevoflurane
Rajan, 2016 [17]	USA	Not mentioned	Not mentioned	Craniotomy (resection of a brain tumour)	68	71	1. DEX: 0.5−1 μg.kg^−1^ 2. Remifentanil: No bolus	1. DEX:0.2–0.7 μg.kg^−1^ 2. Remifentanil: 0.08–0.15 μg.kg^−1^	Propofol + Recoronium + Fentanyl + Sevoflurane
Song, 2016 [18]	China	I-III	18–60	Supratentorial craniotomy	25	27	1. DEX: 0.5 μg.kg^−1^ 2. 0.9% Saline	1. DEX: 0.2–0.5 μg.kg^−1^ 2. 0.9% Saline	Midazolam + Fentanyl + Propofol + Remifentanil + Cisatracurium
Sriganesh, 2019 [2]	India	Not mentioned	18–60	Supratentorial craniotomy	12	12	NO BOLUS	1. DEX: 0.5 μg.kg^−1^ 2. Fentanyl: 1 μg.kg^−1^	Thiopentone + Lignocaine + Vecuronium + Fentanyl + Isoflurane
Turgut, 2009 [19]	Turkey	I-III	18–80	Supratentorial craniotomy	25	25	1. DEX: 1 μg.kg^−1^ 2. Remifentanil: 1 μg.kg^−1^	1. DEX: 0.2–1 μg.kg^−1^ 2. Remifentanil: 0.05μg.kg^−1^	Propofol + Cisatracurium
Yun, 2017 [20]	China	I-II	35–65	Supratentorial craniotomy	D1: 43, D2: 46	45	1. D1: 0.4 μg.kg^−1^ 2. D2: 0.8 μg.kg^−1^ 3. 0.9% Saline	NO CONTINUOUS INFUSION	Pantoprazole + Propofol + Sufentanil + Cisatracurium + Sevoflurane, Remifentanil
Goettel, 2016 [21]	Canada	I-III	18–80	Supratentorial craniotomy (awake procedure)	25	25	1. DEX: 1 μg.kg^−1^ 2. Propofol/Remifentanil: no bolus	1. DEX: 0.2–1 μg.kg^−1^ 2. Propofol/Remifentanil: 25–150 μg.kg^−1^, 0.01–0.1 μg.kg^−1^	Fentanyl + Bupivacaine
Tanskanen, 2006 [22]	Finland	Not mentioned	20–65	Supratentorial craniotomy	35	18	NO BOLUS	1. D1: 0.2 ng.ml^−1^ 2. D2: 0.4 ng.ml^−1^ 3. 0.9% Saline	Fentanyl + Thiopental + Pancuronium + NO + Isoflurane
Günes, 2005 [23]	Turkey	I-II	19–70	Craniotomy (resection of vascular or space-occupying lesions)	39	39	NO BOLUS	1. DEX: 0.6–1.2 mg.kg^−1^ 2. Remifentanil: 0.25 μg.kg^−1^	Propofol + Remifentanil + Vecuronium + NO
Kim, 2016 [24]	South Korea	I-II	20–70	Craniotomy (clipping of unruptured cerebral aneurysm)	32	32	1. DEX: 0.5 μg.kg^−1^ 2. Remifentanil: 0.5 μg.kg^−1^	NO CONTINUOUS INFUSION	Propofol + Remifentanil + Rocuronium + Sevoflurane
Zheng, 2020 [25]	China	I-II	27–59	Craniotomy (intracranial aneurysm)	44	22	NO BOLUS	1. D1: 1 μg.kg^−1^ 2. D2: 0.5 μg.kg^−1^ 3. Control: 0.9% Saline	Midazolam + Sufentanil + Atracurium + Etomidate
Prathapadas, 2020 [26]	India	I-II	18–50	Supratentorial craniotomy	20	20	NO BOLUS	1. DEX: 0.2 μg.kg^−1^ 2. Control: 0.9% Saline	Fentanyl + Propofol + Vecuronium + Sevoflurane

ASA = American Society of Anaesthesiology, DEX = dexmedetomidine, Age (y) = age in years.

**Table 2:** Overview of the reported effects of DEX on postoperative pain in the included trials (DEX group(s) compared to the control group).

References	Favourable Effect of DEX	Reported *p*-Value Intervention vs. Control	Reported Pain Variable and Raw Numbers	Results
Bekker et al. [16]	No	*P* = 0.4151	Opioid consumption	No significant difference between the groups
Peng et al. [7]	Yes	Pain scores: *p* < 0.05 Opioid consumption: *p* < 0.05	Pain scores (VRS), also comments on opioid consumption	Lower pain scores and opioid consumption in the DEX group
Rajan et al. [17]	Yes	Pain scores: *p* < 0.001 Opioid consumption: *p* < 0.001	Pain scores (VAS), also comments on opioid consumption	Lower pain scores and opioid consumption in the DEX group
Song et al. [18]	Yes	Pain scores: *p* < 0.001 Opioid consumption: *p* < 0.001	Pain scores (NRS), also comments on opioid consumption	Lower pain scores and opioid consumption in the DEX group
Sriganesh et al. [2]	No	Pain scores: *p* > 0.05	Pain scores (NRS), also comments on opioid consumption	No significant difference between the groups
Turgut et al. [19]	Yes	Not described	Opioid consumption	Lower opioid consumption in the DEX group
Yun et al. [20]	Yes	Pain scores: Difference between control- and medium-dose DEX group: *p* < 0.05 Difference between control- and small-dose DEX group: *p* < 0.05.	Number of patients having no pain with the lowest pain scores (VRS)	Number of patients without pain greater in DEX group
Goettel et al. [21]	Yes	Pain scores: *p* = 0.026 and 0.031	Pain scores (VAS), also comments on opioid consumption	Lower pain scores and opioid consumption in the DEX group
Tanskanen et al. [22]	No	Not reported	Pain scores (VAS), also comments on opioid consumption	Described as not significant
Gunes et al. [23]	Yes	*p* = 0.013	Opioid consumption	Lower opioid consumption in the DEX group
Kim et al. [24]	No	Pain scores: *p* = 0.57 Opioid consumption: *p* = 0.59	Pain scores (VAS), also comments on opioid consumption	No significant difference in pain scores or opioid consumption between the groups
Zheng et al. [25]	Yes	*p* < 0.05	Pain scores (VAS)	Lower pain scores in DEX groups compared to the control group.
Prathapadas et al. [26]	No	Not reported	Pain scores (VAS)	Pain scores were comparable between the groups

DEX, dexmedetomidine, VAS, Visual Analogue Scale, VRS, Verbal Rating Scale, NRS, Numeric Rating Scale.

## Data Availability

The search string used to acquire the data is provided in the Supplementary Material.

## Supplementary Materials

The following are available online at https://www.mdpi.com/article/10.3390/brainsci11121636/s1, Table S1: GRADE evidence profile of the included trials. File S1: Search string.
