# Supplementary material for: Effects of Intraoperative Dexmedetomidine Infusion on Postoperative Pain after Craniotomy: A Narrative Review
Source: Brain Sci. 2021 Dec 11;11(12):1636. doi: 10.3390/brainsci11121636 (PMC8699313; doi:10.3390/brainsci11121636)
Supplement: Supplementary file 1 [file brainsci-11-01636-s001.zip › Suppl table S1 GRADE table brain science.pdf]

Suppl table S1 GRADE evidence profile of the included trials

References	Participants	Risk of bias	Inconsistency	Indirectness	Imprecision	Publication bias	Quality of evidence
**Bekker et al. [16]**	56	No serious risk of bias	No serious risk of inconsistency	No serious risk of indirectness	No serious risk of imprecision	Undetected	**HIGH**
**Peng et al. [7]**	76	No serious risk of bias	No serious risk of inconsistency	No serious risk of indirectness	No serious risk of imprecision	Undetected	**HIGH**
**Rajan et al. [17]**	139	No serious risk of bias	No serious risk of inconsistency	No serious risk of indirectness	No serious risk of imprecision	Undetected	**HIGH**
**Song et al. [18]**	52	Serious risk of bias*	No serious risk of inconsistency	No serious risk of indirectness	Serious risk of imprecision*	Undetected	**MODERATE**
**Sriganesh et al. [2]**	24	No serious risk of bias	No serious risk of inconsistency	No serious risk of indirectness	No serious risk of imprecision	Undetected	**HIGH**
**Turgut et al. [19]**	50	Serious risk of bias*	No serious risk of inconsistency	No serious risk of indirectness	Serious risk of imprecision*	Undetected	**MODERATE**
**Yun et al. [20]**	134	No serious risk of bias	No serious risk of inconsistency	No serious risk of indirectness	No serious risk of imprecision	Undetected	**HIGH**
**Goettel et al. [21]**	50	Serious risk of bias*	No serious risk of inconsistency	No serious risk of indirectness	Serious risk of imprecision*	Undetected	**MODERATE**
**Tanskanen et al. [22]**	53	No serious risk of bias	No serious risk of inconsistency	No serious risk of indirectness	No serious risk of imprecision	Undetected	**HGH**
**Günes et al. [23]**	78	Serious risk of bias‡	No serious risk of inconsistency	Serious risk of indirectness‡	Serious risk of imprecision‡	Undetected	**LOW**
**Kim et al. [24]**	64	No serious risk of bias	No serious risk of inconsistency	No serious risk of indirectness	No serious risk of imprecision	Undetected	**HIGH**
**Zheng et al. [25]**	66	No serious risk of bias	No serious risk of inconsistency	No serious risk of indirectness	No serious risk of imprecision	Undetected	**HIGH**
**Prathapadas et al. [26]**	40	No serious risk of bias	No serious risk of inconsistency	No serious risk of indirectness	No serious risk of imprecision	No serious risk of imprecision	**HIGH**

* Quality was rated down for risk of bias due to imprecision and inadequately generated randomization sequence and inadequate information about allocation concealment and blinding

† Quality was rated down for risk of bias due to being a retrospective study

‡ Quality was rated down for risk of bias due to completely missing information about the randomization sequence and inadequate information about allocation concealment and blinding
