# Supplementary material for: Effects of Intraoperative Dexmedetomidine Infusion on Postoperative Pain after Craniotomy: A Narrative Review
Source: Brain Sci. 2021 Dec 11;11(12):1636. doi: 10.3390/brainsci11121636 (PMC8699313; doi:10.3390/brainsci11121636)
Supplement: Supplementary file 1 [file brainsci-11-01636-s001.zip › Supplementary file S1.pdf]

Supplementary File

Search String

Ovid Technologies

Search for: 3 and 7 and 11

Results: 185

Database: Ovid MEDLINE(R) and Epub Ahead of Print, In-Process & Other Non-Indexed Citations, Daily and Versions(R) <1946
to October 13, 2021>
Search Strategy:
----
1 Dexmedetomidine/ (3099)
2 Dexmedetomidine.mp. (5253)
3 1 or 2 (5253)
4 Intraoperative Care/ (16326)
5 Intraoperative Period/ (13658)
6 (intraoperative or intra-operative or peroperative or per-operative).ti,ab,kw. (125692)
7 4 or 5 or 6 (139083)
8 Neurosurgery/ (14431)
9 exp Neurosurgical Procedures/ (184443)
10 (neurosurg* or neuro-surg*).ti,ab,kw. (48007)
11 8 or 9 or 10 (222118)
12 3 and 7 and 11 (185)
